# Vibration analysis of nanobeams subjected to gradient-type heating due to a static magnetic field under the theory of nonlocal elasticity

**DOI:** 10.1038/s41598-022-05934-0

**Published:** 2022-02-03

**Authors:** Hijaz Ahmad, Ahmed E. Abouelregal, Moez Benhamed, Maged Faihan Alotaibi, Abir Jendoubi

**Affiliations:** 1 Information Technology Application and Research Center, Istanbul Ticaret University, 34445 Istanbul, Turkey; 2 Department of Mathematics, Faculty of Humanities and Social Sciences, Istanbul Ticaret University, 34445 Istanbul, Turkey; 3 Department of Mathematics, College of Science and Arts, Jouf University, Al-Qurayat, Saudi Arabia; 4 Basic Sciences Research Unit, Jouf University, Al-Qurayat, Saudi Arabia; 5 Department of Mathematics, Faculty of Science, Mansoura University, Mansoura, 35516 Egypt; 6 Department of Mathematics, Faculty of Sciences of Tunis, University of Tunis El Manar LR03ES04, 2092 Tunis, Tunisia; 7 Deanship of Scientific Research, King Abdulaziz University, Jeddah, Saudi Arabia; 8 University of Sherbrooke, Sherbrooke, QC Canada

**Keywords:** Materials science, Mathematics and computing, Nanoscience and technology, Physics

## Abstract

Nanoelectromechanical systems (NEMS) have received great interest from researchers around the world since the advent of nanotechnology and nanoengineering. This can be attributed due to the unique characteristics of NEMS devices and their wide range of applications. Among these applications, nanobeams and nanotubes now have an important role in the design of a variety of NEMS engineering devices. In the current research, the thermoelastic vibration analysis of Euler–Bernoulli nanobeams has been investigated using the theory of non-local elasticity proposed by Eringen. Also to study the effect of temperature change, the generalized thermoelastic model with dual phase-lag (DPL) is applied. The studied nanobeam is subjected to an axial thermal excitation load and surrounded by a magnetic field of constant strength. The Laplace transform technique has been used to solve the system differential equations and to find an approximate analytical solution for the different physical fields of the nanobeam. The numerical results obtained for the studied variables have been graphically clarified and discussed analytically. The effects of various influencing factors such as magnetic field strength, temperature change, non-local parameter as well as ramp type parameter have been examined and studied in detail.

## Introduction

It is known that the energy equation in terms of temperature is a parabola according to the assumptions of the classical Fourier law. As a result, using the classic Fourier law, the temperature spreads at an infinite rate. To address this flaw, several ideas have been developed. Theories of generalized thermoelasticity, or thermoelasticity with the second acoustic effect, are the terms used to describe these ideas^1^. The concept of relaxation time was incorporated into the standard Fourier law by Lord and Shulman as a simple idea^2^. They included the impact of heat flux rate in the flux-temperature relationship and demonstrated that, under such assumptions, the energy equation is a hyperbolic equation, resulting in thermal wave propagation at a limited speed.

Green and Lindsay^3^ proposed the second generalized thermoelasticity theory, which included the temperature rate as a dependent term on two relaxation time factors. In the entropy expression and stress correlation, the authors identified two distinct lag periods. In compared to classical theory, the Green–Lindsay theory (GL) has more extensive energy and motion equations. Green and Naghdi in^4^ used the displacement-temperature-flux rate in Fourier's law to create a novel model that did not incorporate energy wastage. Tzou^5–7^, who provided two separate phase delays in the heat conduction Fourier's law, proposed a generalized thermoelasticity model with dual-phase lag (DPL). The first is for the heat flux vector, while the second is for the temperature gradient. In addition to the preceding models, a number of popular thermal elasticity models have been presented, all of which are based on the theories that have been generalised in a number of articles^8–12^.

Nanoelectromechanical systems (NEMS) have garnered a lot of interest from researchers all over the globe since the emergence of nanotechnology and nano-engineering. This is due to NEMS devices' unique characteristics and wide range of applications^13^. Nanobeams and nanotubes are now possible design possibilities in many engineering NEMS devices at the nanoscale scale^14^, and they are playing significant roles in many engineering NEMS devices. Due to their unique properties and functions, micro/nanoelectromechanical systems (MEMS and NEMS) have been of great research interest. These systems are widely used in many areas of technology and engineering, including vehicles and electronics^15^. Some micro and nano structures, as well as length and width differences that have a significant impact on the properties and performance of those structures, may have geometric irregularities during production, assembly and packaging. It is well known that the mechanical response of nanostructures is of large dimensions, thus small effects on the mechanical behavior of nanostructures must be considered^16^.

Classical continuum theories are well known because they are not able to correctly estimate the experimentally detected size-dependent behavior of small-scale structures; any sizes on the order of microns and sub-microns. To solve this problem, a few more general ordinary continuum mechanics theories have been developed and used to record size-dependent behavior involving additional physical constants and conventional Lamé constants. Some of these theories are the strain gradient^17,18^, modified strain gradient^19^, couple stress^2,20^, modified couple stress^21^, and the theory of nonlocal continuum^22,23^. In nano-mechanical applications including distribution of elastic waves via grid, composite wave propagation, dislocating dynamics and mechanical broken waves, surface fluids, etc., theory of non-local elasticity was used. In all nanostructures, the mechanical conduct of the nano-tubes and nanobeams was studied most often. The fundamental difference between the conventional Eringen's nonlocal theory of elasticity is based on the definition of stress^24–29^.

There is a growing interest among scientists in using Eringen's nonlocal elasticity differential model^22,23^. For this reason, the state of stress at one location in the body is determined by the states of strain at all other sites in the body, according to the theory of non-local elasticity. This is in stark contrast to the traditional continuum mechanics theory, which states that the stress state at a given locus depends only on the strain state at the same locus. The theory of non-local elasticity has been widely used to explore nanomechanical applications such as nanostructure vibrations and wave propagation, fluid-loaded nano-device stability, dislocation mechanics, crack mechanics, etc.^30^.

Using simple one-dimensional beam models, Challamel^31^ considered the self-adjointness of Eringen's nonlocal elasticity theory. It is demonstrated that Eringen's model may not be self-adjoint and that raising Eringen's small length scale coefficient can result in an unanticipated stiffening effect for a cantilever's fundamental vibration frequency. This is clearly inconsistent with all other boundary condition softening results, as well as the higher vibration modes of a cantilever beam. Eringen's nonlocal elasticity has recently been proven to be a good fit for microstructured (discrete) media^31^. Given that discrete media is a conservative system at the local level, a conservative macro-scale is logical (conservative nonlocal elasticity equivalent continuum, or self-adjoint macroscopic system). The softening phenomena generated by the small length scale effect is best described by Eringen's corrected nonlocal elasticity with self-adjoint boundary conditions, which cannot be attained with nonself-adjoint Eringen elasticity^31^. As a result, inside the micromechanics-based energy arguments, we recommend using differential equations with the specified associated boundary conditions. More details on this point can be referred to in^32–36^.

We therefore recommend the use of the differential equations with the proposed associated boundary conditions within the micromechanics-based energy arguments.

According to the classical nonlocal elasticity, the stresses at a given point of a continuum medium depend on the strains evaluated not only at the point itself but also in the overall volume. Clearly, this general integral constitutive relation leads to sophisticated integro-differential equations, which are challenging even for 1D problems. Therefore, Eringen proposed a simplified differential form, which may be considered as equivalent to general integral formulation problems, e.g. propagation of plane waves in an unbounded medium. Over the last fifteen years, this differential formulation has been often employed as a framework for problems instability and vibrations of nano- beams, plates, and shells, as well as carbon nanotubes.

Among the excellent research mentioned above, only a few thermoelastic models were employed to study the thermal behavior of the nanobeam system, and they mostly focused on the linear frequency of free vibrations. According to past research and the author's expertise, the thermoelastic behavior of the nanobeam has not been investigated yet using generalized thermoelasticity models with phase lags. The thermoelastic vibration of nonlocal nanobeams subjected to a ramp-type thermal load and a longitudinal magnetic field is examined analytically for the first time when the nanobeam is simply supported in the current study.

The thermoelastic vibration and mechanical behaviors of an Euler–Bernoulli nanobeam under thermal and magnetic environments are investigated in the following investigation due to the increasing use of nanostructures in nanotechnology and nano-devices Maxwell's equations are used to calculate the transverse Lorentz force due to the horizontal magnetic field vector. The analytical solutions of the normal deflections, temperature, and bending moments for Euler–Bernoulli nanobeams are obtained specifically with several unknown constants using the Laplace transformation. The thermoelastic responses of nanobeams are numerically investigated with the influences of a nonlocal parameter, a ramp-type parameter, and a magnetic field. For forced vibration responses of nanobeams, the variations between classical coupled theory and generalized thermoelasticity are also studied.

## Governing basic equations

As is evident from Eringen's non-local elasticity theory, stress at any point in the body depends on current stresses as well as strains elsewhere in the body. The dynamics and some experimental results of acoustic scattering are used in atomic network theory to explain Eringen's non-local elasticity. The integral space, which describes the weighted averages of the pressure tensor of a related body point, is one of the basic equations in this theory. The theory introduces the effect of small scale in the integral constitutive relationship of space.

For homogeneous and isotropic elastic solids, the non-local elasticity equation are^22,23^

1 $$\sigma _{{kl}} = \int_{v} {\alpha \left( {\left| {r - r^{\prime } } \right|,\chi } \right)\tau _{{kl}} \left( {r^{\prime } } \right)dV\left( {r^{\prime } } \right)}$$

where $V$ is the volume of the elastic body and $\sigma_{kl}$ denotes the nonlocal stresses tensor, $\left| {r - r^{\prime}} \right|$ indicates the Euclidean distance. The effect of the strain at point $r^{\prime}$ on the stress at point $r$ in the elastic body is described by the nonlocal kernel $\alpha \left( {\left| {r - r^{\prime}} \right|} \right)$. The value of $\chi$ depends on the ratio $e_{0} a/l$ which is material constant. The internal characteristic length is $a$, the external characteristic length is $l$, and the constant $e_{0}$ is specific to each material.

The local stress tensor $\tau_{kl} \left( {r^{\prime}} \right)$ may be written^22^

2 $$\tau_{kl} \left( {r^{\prime}} \right) = \lambda \varepsilon_{mm} \left( {r^{\prime}} \right)\delta_{kl } + \mu \varepsilon_{kl } \left( {r^{\prime}} \right) - \gamma \delta_{kl } \theta \left( {r^{\prime}} \right).$$

In the above relation, $\theta = T - T_{0}$ is the excess temperature, in which $T_{0}$ is the environmental temperature, $\lambda$ and $\mu$ being Lamé's constants, $\gamma = \alpha_{t} \left( {3\lambda + 2\mu } \right) = \alpha_{t } E/\left( {1 - 2\nu } \right)$, $\alpha_{t}$ symbolizes the thermal expansion coefficient, $E$ denotes Young modulus,$\nu$ designates Poisson's ratio,$\delta_{ij}$ is Kronecker delta function, $\varepsilon_{mm} \left( {x^{\prime}} \right)$ describes the strain tensor which is given by

3 $$\varepsilon_{{kl{ }}} \left( {r^{\prime}} \right) = \frac{1}{2}\left( {\frac{{\partial u_{k} \left( {r^{\prime}} \right)}}{{\partial r^{\prime}_{1} }} + \frac{{\partial u_{1} \left( {r^{\prime}} \right)}}{{\partial r^{\prime}_{k} }}} \right),$$

where $u_{l} (r^{^{\prime}} )$ is the vector of displacement at the point $x^{\prime}$ in the body.

The following are some of the intriguing properties of the nonlocal modulus $\alpha \left( {\left| {r - r^{\prime}} \right|} \right)$ ^23^:

- It reaches its peak at $r = r^{\prime}$ attenuating with $\left| {r - r^{\prime}} \right|$.
- Also, when $\tau = e_{0} a/l \to 0$, we thus expect that $\alpha$ is a delta sequence i.e. $\mathop {\lim }\limits_{\tau \to 0} \alpha \left( {\left| {r - r^{\prime}} \right|,\chi } \right) = \delta \left( {\left| {r - r^{\prime}} \right|} \right)$.
- In addition, nonlocal theory should be able to approximate atomic lattice dynamics for small intrinsic characteristic lengths, i.e. when $\tau = 1$.

When the kernel $\alpha$ is chosen as^23,37^:

4 $$\alpha \left( {\left| {r - r^{\prime } } \right|,\chi } \right) = \frac{1}{{2\pi \chi ^{2} l^{2} }}K_{0} \left( {\frac{{\left\| {r - \mathop {r^{ {\prime }} }\nolimits^{ {\prime }} } \right\|}}{{\chi l}}} \right),$$

where $K_{0}$ is the modified Bessel function, Eq. (1) may be simplified as

5 $$\left( {1 - \left( {ae_{0} } \right)^{2} \nabla^{2} } \right)\sigma_{kl} = \tau_{ij} .$$

Tzou in^5–7^ provides a simplified version of the classical thermoelastic model, in which the Fourier law is replaced by an approximation of the equation

6 $${\varvec{q}}\left( {r,t + \tau_{q} } \right) = - K\nabla \theta \left( {r,t + \tau_{\theta } } \right),$$

where ${\varvec{q}}{ }$ indicates the heat flow, $K$ symbolizes the thermal conductivity, $\tau_{\theta }$ is the phase lag of the heat flow (PLH), and $\tau_{q}$ is the phase lag of gradient of temperature (PLT).

According to Eq. (6), the temperature gradient $\nabla \theta$ generated across a material volume at position $r$ at time $t + \tau_{\theta }$ results in a heat flux ${\varvec{q}}$ to flow at a different instant of time $t + \tau_{q}$. This delay is frequently explained in terms of the material's microstructure. We direct the reader to Tzou's^5–7^ work to clarify the applicability of this type of theory.

We can make use of truncated Taylor expansions to replace Eq. (6) in Tzou's theorem or its extension^6^. The following equation can be used to approximate the previous equation:

7 $$\left( {1 + \tau_{q } \frac{\partial }{\partial t} + \frac{{\tau_{q}^{2} }}{2}\frac{{\partial^{2} }}{{\partial t^{2} }}} \right){\varvec{q}} = - K\nabla \left( {1 + \tau_{\theta } \frac{\partial }{\partial t}} \right)\theta ,{ }0 \le \tau_{\theta } < \tau_{q} .$$

The heat flux ${\varvec{q}}\left( {r,t + \tau_{q} } \right)$ has been enlarged to second order, and the temperature gradient $\nabla \theta \left( {r,t + \tau_{\theta } } \right)$ has been expanded to first order. It is also possible to propose a higher-order thermoelasticity theory. Equation (7) describes the Cattaneo heat transfer model for $\tau_{\theta }$, and simplifies to the classical Fourier law when $\tau_{q } = \tau_{\theta } = 0$.

The generalized DPL heat transfer equation proposed by Tzou^5,6^ is given by

8 $$K\left( {1 + \tau_{\theta } \frac{\partial }{\partial t}} \right)\nabla^{2} \theta = \left( {1 + \tau_{q } \frac{\partial }{\partial t} + \frac{{\tau_{q}^{2} }}{2}\frac{{\partial^{2} }}{{\partial t^{2} }}} \right)\left( {\rho C_{E } \frac{\partial \theta }{{\partial t}} + \gamma T_{0 } \frac{\partial e}{{\partial t}} - \rho Q} \right),$$

where $C_{E}$ is the specific heat, $e = \frac{\partial u}{{\partial x}} + \frac{\partial v}{{\partial y}} + \frac{\partial w}{{\partial z}}$ is the volumetric strain and $Q$ is the heat source.

## Maxwell’s relations

Due to the application of an initial magnetic field ${\varvec{H}}$, both the magnetic field ${\varvec{h}}$ and the electric field ${\varvec{E}}_{l}$ are induced. Below are the basic linear electrodynamic equations of a slow-moving medium for the ideal uniform electrical conductivity of an elastic solid (neglecting the load density)^32^

9 $${\varvec{J}} = \nabla \times {\varvec{h}},\;{ }\nabla \times {\varvec{E}}_{l} = - \mu_{0} \frac{{\partial {\varvec{h}}}}{\partial t},\;{ }{\varvec{E}}_{l} = - \mu_{0} \left( {\frac{{\partial {\varvec{U}}}}{\partial t} \times {\varvec{H}}} \right),\;{\varvec{h}} = \nabla \times \left( {{\varvec{U}} \times {\varvec{H}}} \right),\;{ }\nabla \cdot {\varvec{h}} = 0,$$

where $\nabla$ is the Hamilton arithmetic operator (nabla) and $\mu_{0}$ denotes the magnetic permeability. Let the displacement vector ${\varvec{U}} = \left( {u,\nu ,{\text{w}}} \right)$, and a longitudinal magnetic field vector ${\varvec{H}} = \left( {H_{x} ,0,0} \right)$, then we have

10 $$\begin{array}{*{20}c} {{\varvec{h}} = H_{x} \left( { - \left( {\frac{\partial v}{{\partial y}} + \frac{\partial w}{{\partial z}}} \right),\frac{\partial v}{{\partial x}},{ }\frac{\partial w}{{\partial x}}} \right)} \\ {{\varvec{J}} = H_{x} \left( { - \left( { - \frac{{\partial^{2} v}}{\partial x\partial z} + \frac{{\partial^{2} w}}{\partial x\partial z}} \right), - \left( {\frac{{\partial^{2} v}}{\partial y\partial z} + \frac{{\partial^{2} w}}{{\partial x^{2} }} + \frac{{\partial^{2} w}}{{\partial z^{2} }}} \right),\left( {\frac{{\partial^{2} w}}{\partial y\partial z} + \frac{{\partial^{2} v}}{{\partial x^{2} }} + \frac{{\partial^{2} v}}{{\partial y^{2} }}} \right)} \right)} \\ \end{array} { }$$

The Lorentz force ${\varvec{F}} = \left( {f_{x} ,f_{y} ,f_{z} } \right)$ is created from the length of the magnetic field and is given by:

11 $${\varvec{F}} = \mu_{0} \left( {{\varvec{J}} \times {\varvec{H}}} \right) = \mu_{0} H_{x}^{2} \left( {0, - \left( {\frac{{\partial^{2} w}}{\partial y\partial z} + \frac{{\partial^{2} v}}{{\partial x^{2} }} + \frac{{\partial^{2} v}}{{\partial y^{2} }}} \right),\left( {\frac{{\partial^{2} v}}{\partial y\partial z} + \frac{{\partial^{2} w}}{{\partial x^{2} }} + \frac{{\partial^{2} w}}{{\partial y^{2} }}} \right)} \right){ }$$

## Formulation of problem

**Figure 1:**
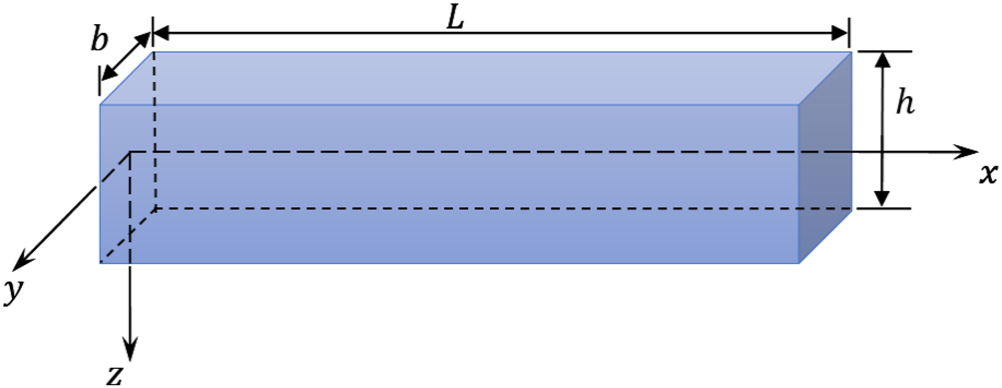
schematic diagram of the nanobeam.

Consider slight bending thin nanobeam with sizes $L\left( {0 \le x \le L} \right)$, thickness $h\left( { - h/2 \le z \le + h/2} \right)$ and width $b\left( { - b/2 \le y \le + b/2} \right)$ (see Fig. 1). We set the $x$-axis along the beam axis and the $y$ and $z$-axes match width and thickness. The nanobeam has small amplitude bending motions along the $x$-axis so that the deflection is consistent with the linear principle of Euler–Bernoulli. his implies that every plane cross-section is perpendicular to the beam axis, flat, and perpendicular to the neutral surface during curvature.

The displacements are therefore are given by

12 $$u = - z\frac{\partial w}{{\partial x}},{ }\nu = 0{ },{ }w\left( {x,y,z,t} \right) = w\left( {x,t} \right).$$

where $w$ is the lateral deflection. The Lorentz force in $z$ direction is written as

13 $$f_{z} = \mu_{0} H_{x}^{2} \frac{{\partial^{2} w}}{{\partial x^{2} }}$$

The non-local constitutive Eq. (5) can be written as^22^:

14 $$\sigma _{x} - \xi \frac{{\partial ^{2} \sigma _{x} }}{{\partial x^{2} }} = - E\left( {z\frac{{\partial ^{2} w}}{{\partial x^{2} }} + \alpha _{T} \theta } \right),$$

where $\sigma_{x}$ is the nonlocal axial stress, $\alpha_{T} = \alpha_{t} /\left( {1 - 2\nu } \right)$ and $\xi = \left( { e_{0} a} \right)^{2}$ is the nonlocal parameter. The equilibrium conditions of the Euler–Bernoulli beam can be described as the transverse nanoscale vibrations described by the equation.

The equilibrium conditions of the Euler–Bernoulli beam can be described as the transverse nanoscale vibrations which can be described by the equation

15 $$\frac{{\partial^{2} M}}{{\partial x^{2} }} + f\left( x \right) = \rho A\frac{{\partial^{2} w}}{{\partial t^{2} }}$$

where $f\left( x \right)$ is the force per length application of an initial magnetic field and $A = bh$.

Bending moment with the help of Eqs. (14) and (15) can achieve the following equation

16 $$M\left( {x,t} \right) - \xi \frac{{\partial^{2} M}}{{\partial x^{2} }} = - EI\left( {\frac{{\partial^{2} w}}{{\partial x^{2} }} + \alpha_{T} { }M_{T} } \right)$$

where $I = bh^{3} /12$ is the cross-sectional inertia moment and $M_{T}$ is the thermal moment

17 $$M_{T} = \frac{12}{{h^{3} }} \mathop \int \limits_{ - h/2}^{h/2} \theta \left( {x,z,t} \right)z dz$$

From Eqs. (15) and (16), we get the equation of motion governing the nanobeam system as

18 $$\frac{{\partial^{4} w}}{{\partial x^{4} }} + \frac{\rho A}{{EI}}\frac{{\partial^{2} w}}{{\partial t^{2} }} - \xi \frac{\rho A}{{EI}}\frac{{\partial^{4} w}}{{\partial t^{2} \partial x^{2} }} - \frac{1}{EI} f\left( x \right) + \frac{\xi }{EI} \frac{{\partial^{2} f\left( x \right)}}{{\partial x^{2} }} + \alpha_{T} \frac{{\partial^{2} M_{T} }}{{\partial x^{2} }} = 0$$

The force per length $f\left( x \right)$ can be obtained by

19 $$f\left( x \right) = Af_{z} = A\mu_{0} H_{x}^{2} \frac{{\partial^{2} w}}{{\partial x^{2} }}$$

In addition, the flexure moment $M\left( {x,t} \right)$ can be expressed as

20 $$M\left( {x,t} \right) = \xi \rho A\frac{{\partial^{2} w}}{{\partial t^{2} }} - \xi A\mu_{0} H_{x}^{2} \frac{{\partial^{2} w}}{{\partial x^{2} }} - EI\left( {\frac{{\partial^{2} w}}{{\partial x^{2} }} + \alpha_{T} M_{T} } \right)$$

Inserting Eq. (29) into Eq. (18), then we have

21 $$\frac{{\partial^{4} w}}{{\partial x^{4} }} + \frac{\rho A}{{EI}}\frac{{\partial^{2} w}}{{\partial t^{2} }} - \xi \frac{\rho A}{{EI}}\frac{{\partial^{4} w}}{{\partial t^{2} \partial x^{2} }} - \frac{{A\mu_{0} H_{x}^{2} }}{EI}\frac{{\partial^{2} w}}{{\partial x^{2} }} + \frac{{\xi A\mu_{0} H_{x}^{2} }}{EI}{ }\frac{{\partial^{4} w}}{{\partial x^{4} }} + \alpha_{T} \frac{{\partial^{2} M_{T} }}{{\partial x^{2} }} = 0$$

The DPL heat equation without the heat source $\left( {Q = 0} \right)$ can be written as follows

22 $$K\left( {1 + \tau_{\theta } { }\frac{\partial }{\partial t}} \right)\left( {\frac{{\partial^{2} \theta }}{{\partial x^{2} }} + { }\frac{{\partial^{2} \theta }}{{\partial z^{2} }}} \right) = \left( {1 + \tau_{q } \frac{\partial }{\partial t} + \frac{{\tau_{q}^{2} }}{2}\frac{{\partial^{2} }}{{\partial t^{2} }}} \right)\left( {\rho C_{{E{ }}} { }\frac{\partial \theta }{{\partial t}} - \gamma T_{{0{ }}} z{ }\frac{\partial }{\partial t}\left( {\frac{{\partial^{2} w}}{{\partial x^{2} }}} \right)} \right)$$

## Analytical solution

The upper and lower sides of the beam have no heat flow (thermally insulated), That's how it is $\frac{\partial \theta }{{\partial z}} = 0$ at $z = \frac{ \pm h}{2}$. For the very thin beam, assuming that the temperature increases vary according to the $\sin \left( {pz} \right)$ function along the thickness direction (sinusoidal anisotropy), where,

23 $$\theta \left( {x,y,z} \right) = {\Phi }\left( {x,t} \right)\sin \left( {pz} \right),p = \frac{\pi }{h}.$$

Thus, Eqs. (20) and (21) become

24 $$\frac{{\partial^{4} w}}{{\partial x^{4} }} + \frac{\rho A}{{EI}}\frac{{\partial^{2} w}}{{\partial t^{2} }} - \xi \frac{\rho A}{{EI}}\frac{{\partial^{4} w}}{{\partial t^{2} \partial x^{2} }} - \frac{{A\mu_{0} H_{x}^{2} }}{EI}\frac{{\partial^{2} w}}{{\partial x^{2} }} + \frac{{\xi A\mu_{0} H_{x}^{2} }}{EI}{ }\frac{{\partial^{4} w}}{{\partial x^{4} }} + \frac{{24\alpha_{T} }}{{h\pi^{2} }}\frac{{\partial^{2} {\Phi }\left( {x,t} \right)}}{{\partial x^{2} }}0{ }$$

25 $$M\left( {x,t} \right) = \xi \left( {\rho A\frac{{\partial^{2} w}}{{\partial t^{2} }} - A\mu_{0} H_{x}^{2} \frac{{\partial^{2} w}}{{\partial x^{2} }}} \right) - EI\left( {\frac{{\partial^{2} w}}{{\partial x^{2} }} + \frac{{24\alpha_{T} }}{{h\pi^{2} }}{{ \Phi }}} \right)$$

Multiplying Eq. (22) by means of $12z/h^{3}$ and integrating it with respect to $z$ through the thickness of the nanobeam from $- h/2$ to $+ h/2$, we get

26 $${ }\left( {1 + \tau_{\theta } { }\frac{\partial }{\partial t}} \right)\left( {\frac{{\partial^{2} \theta_{{1{ }}} }}{{\partial x^{2} }} - { }\frac{{\pi^{2} }}{{h^{2} }}{{ \Phi }}} \right) = \left( {1 + \tau_{q } \frac{\partial }{\partial t} + \frac{{\tau_{q}^{2} }}{2}\frac{{\partial^{2} }}{{\partial t^{2} }}} \right)\left( {\frac{{\rho C_{E } }}{K}{ }\frac{{\partial {\Phi }}}{\partial t} - \frac{{\gamma T_{{0{ }}} \pi^{2} h}}{24K}{ }\frac{\partial }{\partial t}\left( {\frac{{\partial^{2} w}}{{\partial x^{2} }}} \right)} \right)$$

In order to simplicity the basic equation, the following non-dimensional quantities are considered:

27 $$\begin{array}{*{20}c} {\left( {x^{\prime},L^{\prime},u^{\prime},w^{'} ,z^{\prime},h^{\prime},b^{\prime}} \right) = \eta c\left( {x,L,u,w,z,h,b} \right),~~~~\xi ^{'} = \eta ^{2} c^{2} \xi ,~~\Phi ^{\prime } = \frac{\Phi }{{T_{{0~}} }},~~} \\ {~\left( {t,t_{0}^{'} ,\tau _{q}^{ {\prime }} ,\tau _{\theta }^{ {\prime }} } \right) = ~\eta c^{2} \left( {t,t_{0} ,\tau _{q} ,\tau _{\theta } } \right),~~\sigma _{x}^{'} = \frac{{\sigma _{{x~}} }}{E},~~~M^{\prime} = \frac{M}{{\eta cEI}}~,\eta = \frac{{\rho C_{{E~}} }}{K},c = \sqrt {\frac{E}{\rho }} .} \\ \end{array}$$

The governing equations are therefore simplified as (dropping the primes)

28 $$\frac{{\partial^{4} w}}{{\partial x^{4} }} + A_{1 } \frac{{\partial^{2} w}}{{\partial t^{2} }} - A_{2 } \frac{{\partial^{4} w}}{{\partial t^{2} \partial x^{2} }} - a_{0 } A_{3 } \frac{{\partial^{2} w}}{{\partial x^{2} }} + a_{0 } A_{4 } \frac{{\partial^{4} w}}{{\partial x^{4} }} + A_{5 } \frac{{\partial^{2} {\Phi }}}{{\partial x^{2} }} = 0$$

29 $${ }\left( {1 + \tau_{\theta } { }\frac{\partial }{\partial t}} \right)\left( {\frac{{\partial^{2} \theta_{{1{ }}} }}{{\partial x^{2} }} - { }A_{{4{ }}} {{ \Phi }}} \right) = \left( {1 + \tau_{q } \frac{\partial }{\partial t} + \frac{{\tau_{q}^{2} }}{2}\frac{{\partial^{2} }}{{\partial t^{2} }}} \right)\left( {\frac{{\partial \theta_{{1{ }}} }}{\partial t} - A_{5} \frac{\partial }{\partial t}\left( {\frac{{\partial^{2} w}}{{\partial x^{2} }}} \right)} \right)$$

30 $$M\left( {x,t} \right) = A_{3 } \frac{{\partial^{2} w}}{{\partial t^{2} }} - \left( {1 + A_{3 } a_{0 } } \right)\frac{{\partial^{2} w}}{{\partial x^{2} }} - A_{2 } {\Phi }\left( {x,t} \right)$$

where

31 $$\begin{array}{*{20}l} {A_{1} = \frac{{12}}{{c^{2} \eta ^{2} h^{2} }},~~A_{2} = \frac{{12\xi }}{{h^{2} }},~~A_{3} = \frac{{12c^{2} }}{{\eta ^{2} h^{2} }}~,~~~A_{4} = \frac{{12\xi }}{{c^{2} h^{2} }},} \\ {A_{5} = \frac{{24T_{{0~}} \alpha _{T} }}{{h\eta c\pi ^{2} }},~~A_{6} = \alpha _{T} T_{{0~}} ,~~a_{{0~}} = \frac{{\mu _{0} H_{x}^{2} }}{\rho }~.~~~} \\ \end{array}$$

## Initial and boundary conditions

It is important to emphasize that both initial conditions and boundary conditions must be considered in order to solve the problem. Suppose that our problem is homogeneous in the initial conditions. First, the initial requirements are considered

32 $$\left. {w\left( {x,t} \right)} \right|_{t = 0} = 0 = \left. {\frac{{\partial w\left( {x,t} \right)}}{\partial t}} \right|_{t = 0} , \left. {{\Phi }\left( {x,t} \right)} \right|_{t = 0} = 0 = \left. {\frac{{\partial {\Phi }\left( {x,t} \right)}}{\partial t}} \right|_{t = 0} .$$

The following boundary conditions can be taken into account. We will assume that the nanobeam is simply supported at the two ends

33 $$\left. {w\left( {x,t} \right)} \right|_{x = 0,L} = 0, \left. {\frac{{\partial^{2} w\left( {x,t} \right)}}{{\partial x^{2} }}} \right|_{x = 0,L} = 0.$$

We also consider, that the first end $x = 0$ of the nanobeam is thermally loaded by ramp-type varying heat. In this case, then we have following boundary condition

34 $$\left. {{\Phi }\left( {x,t} \right)} \right|_{x = 0} = {\Phi }_{0} \left\{ {\begin{array}{*{20}c} {0 , t \le 0,} \\ {\frac{t}{{t_{0 } }}, 0 \le t \le t_{0 } } \\ {1, t > t_{0 } ,} \\ \end{array} } \right.$$

where the ramp-type parameter is denoted by $t_{0}$ and ${\Phi }_{0}$ is a constant.

Furthermore, at the end side $x = L$ of the nanobeam is thermally insulated. Then we have

35 $$\frac{{ \partial {\Phi }}}{\partial x} = 0 , x = L.$$

## Solution of the problem

Applying the Laplace transform to Eqs. (28)–(30), then we have

36 $$\begin{array}{*{20}c} {{ }\left( {{ }\frac{{d^{4} }}{{dx^{4} }} - B_{1} \frac{{d^{2} }}{{dx^{2} }} + B_{2} } \right)\overline{w} = - B_{3} \frac{{d^{2} {\overline{\Phi }}_{{1{ }}} }}{{dx^{2} }},{ }} \\ {\frac{{d^{2} {\overline{\Phi }}_{{1{ }}} }}{{dx^{2} }} - B_{{4{ }}} {\overline{\Phi }} = q{\overline{\Phi }}_{{1{ }}} - B_{{5{ }}} q{ }\frac{{d^{2} \overline{w}}}{{dx^{2} }}{ }} \\ \end{array} { }$$

37 $$\overline{M}\left( {x,t} \right) = A_{3} s^{2} \overline{w} - \left( {1 + a_{0 } A_{3} } \right)\frac{{d^{2} \overline{w}}}{{dx^{2} }} - A_{2} {\overline{\Phi }}$$

By elimination of ${\overline{\Theta }}$ or $\overline{w}$ from Eq. (36), we infer that

38 $$\left( {\left( {D^{2} - B_{4 } )(D^{4} - B_{1 } D^{2} + B_{2} } \right) - B_{5 } B_{3 } D^{2} } \right)\left\{ {{\overline{\Phi }},\overline{w}} \right\}\left( x \right) = 0$$

with

39 $$\begin{array}{*{20}c} { B_{1 } = \frac{{\left( {A_{2} s^{2} + a_{0 } A_{3 } } \right)}}{{\left( {1 + a_{0 } A_{4 } } \right)}} , B_{2 } = \frac{{A_{1} s^{2} }}{{\left( {1 + a_{0 } A_{4 } } \right)}} , B_{3 } = \frac{{A_{5} }}{{\left( {1 + a_{0 } A_{4 } } \right)}}, } \\ { B_{4 } = A_{4 } + q, B_{5 } = qA_{4 } , q = \frac{{1 + s\tau_{q } + s^{2} \frac{{\tau_{q}^{2} }}{2}}}{{1 + \tau_{\theta } { }s}}. } \\ \end{array}$$

Equation (38) can be expressed as

40 $$\left( {D^{6} - AD^{4} + BD^{2} - C} \right)\left\{ {\overline{\theta }_{1 } ,\overline{w}} \right\}\left( x \right) = 0,$$

where

41 $$A = B_{1} + B_{4} + B_{5} B_{3} , B = B_{1} B_{4} + B_{2} , C = B_{2} B_{4 }$$

Introducing $m_{i} \left( {i = 1,2,3} \right)$ into Eq. (40), one gets

42 $$\left[ {\left( {D^{2} - m_{1}^{2} } \right)\left( {D^{2} - m_{2}^{2} } \right)\left( {D^{2} - m_{3}^{2} } \right)} \right]\left\{ {{\overline{\Phi }},\overline{w}} \right\} = 0,$$

where $D = d/dx$ and $m^{2}_{1 } , m^{2}_{2 }$ and $m^{2}_{3 }$ are the roots of the characteristic equation

43 $${ }m^{6} - Am^{4} + Bm^{2} + C = 0$$

The roots of Eq. (43) satisfy the well-known relations:

44 $$m_{1}^{2} + \;m_{2}^{2} + \;m_{2}^{2} = A,\,\,\,\,m_{1}^{2} \;m_{2}^{2} + \;m_{2}^{2} m_{3}^{2} + m_{3}^{2} m_{1}^{2} = B,\,\,\,\,\,m_{1}^{2} \;m_{2}^{2} \;m_{2}^{2} = C$$

These roots are given by

45 $$\begin{array}{*{20}c} {m^{2}_{1 } = \frac{1}{3}\left[ {\left( {2p_{0} \sin q_{0} } \right) + A} \right],} \\ { m^{2}_{2 } = - \frac{1}{3}p_{0} \left[ {\sqrt 3 \cos (q_{0} ) + \sin (q_{0} )} \right] + \frac{1}{3}A,} \\ {m^{2}_{3 } = \frac{1}{3}p_{0} \left[ {\sqrt 3 \cos (q_{0} ) + \sin (q_{0} )} \right] + \frac{1}{3}A,} \\ {p_{0} = \sqrt {A^{2} - 3B} , q_{0} = \frac{1}{3}\sin^{ - 1} \left( { - \frac{{2A^{3} - 9AB + 27C}}{{2p^{3}_{0 } }}} \right).} \\ \end{array}$$

The solution of Eq. (42) can be represented by

46 $$\overline{w}\left( {x,s} \right) = \mathop \sum \limits_{i = 1}^{3} \left( {C_{i} {\text{e}}^{{ - m_{i} x}} + C_{i + 3} {\text{e}}^{{m_{i} x}} } \right){ }$$

47 $${\overline{\Phi }}\left( {x,s} \right) = \mathop \sum \limits_{i = 1}^{3} \left( {\beta_{i} C_{i} {\text{e}}^{{ - m_{i} x}} + \beta_{i + 3} C_{i + 3} {\text{e}}^{{m_{i} x}} } \right)$$

where $\beta_{i}$ and $C_{i}$ are constant coefficients depending on $s$ and $\beta_{i} { } = - \frac{{B_{5} m^{2}_{i} }}{{m^{2}_{i} - B_{4} }}$.

From Eqs. (23) and (47), the temperature in the transformed domain is given by

48 $$\overline{\theta }\left( {x,z,s} \right) = \sin \left( {pz} \right)\mathop \sum \limits_{i = 1}^{3} \left( {\beta_{i} C_{i} {\text{e}}^{{ - m_{i} x}} + \beta_{i + 3} C_{i + 3} {\text{e}}^{{m_{i} x}} } \right)$$

Using the above equations, the bending moment $\overline{M}$ represented in Eq. (37) can be given by

49 $$\overline{M} = - \mathop \sum \limits_{I = 1}^{3} \left( {\left( {1 + a_{0 } A_{3} } \right)m^{2}_{{i{ }}} - A_{3} s^{2} + A_{2} \beta_{i} } \right)\left( {C_{i} e^{{ - m_{i} x}} + C_{i + 3} e^{{m_{i} x}} } \right)$$

In addition, after using Eq. (12), the axial displacement $\overline{u}$ will be in the form

50 $$\overline{u} = - z\frac{{d\overline{w}}}{dx} = z\mathop \sum \limits_{i = 1}^{3} m_{i} \left( {C_{i} e^{{ - m_{i} x}} - C_{i + 3} e^{{m_{i} x}} } \right)$$

In addition, the strain $\overline{e}$ will be

51 $$\overline{e} = \frac{{d\overline{u}}}{dx} = - z\mathop \sum \limits_{i = 1}^{3} m^{2}_{{i{ }}} \left( {C_{i} e^{{ - m_{i} x}} + C_{i + 3} e^{{m_{i} x}} } \right)$$

The boundary conditions after using Laplace transform can be written as

52 $$\left. {\overline{w}\left( {x,s} \right)} \right|_{x = 0,L} = 0, \left. {\frac{{d^{2} \overline{w}\left( {x,s} \right)}}{{dx^{2} }}} \right|_{x = 0,L} = 0$$

53 $$\left. {{\overline{\Phi }}\left( {x,s} \right)} \right|_{x = 0} = {\Phi }_{0} \left( {\frac{{1 - e^{{ - t_{0} s}} }}{{t_{0} s^{2} }}} \right) = \overline{G}\left( s \right)$$

54 $$\frac{{{ }\partial {\overline{\Phi }}}}{\partial x} = 0{ },{ }x = L$$

Using the above conditions, six linear equations are given as follows in matrix format

55 $$\left[ {\begin{array}{*{20}c} 1 & 1 & 1 & 1 & 1 & 1 \\ {e^{{ - m_{1} L}} } & {e^{{ - m_{2} L}} } & {e^{{ - m_{3} L}} } & {e^{{m_{1} L}} } & {e^{{m_{2} L}} } & {e^{{m_{3} L}} } \\ {m_{1}^{2} } & {m_{2}^{2} } & {m_{3}^{2} } & {m_{1}^{2} } & {m_{2}^{2} } & {m_{3}^{2} } \\ {m_{1}^{2} e^{{ - m_{1} L}} } & {m_{2}^{2} e^{{ - m_{2} L}} } & {m_{3}^{2} e^{{ - m_{3} L}} } & {m_{1}^{2} e^{{m_{1} L}} } & {m_{2}^{2} e^{{m_{2} L}} } & {m_{3}^{2} e^{{m_{3} L}} } \\ {\beta_{1} } & {\beta_{2} } & {\beta_{3} } & {\beta_{1} } & {\beta_{2} } & {\beta_{3} } \\ { - m_{1} \beta_{1} e^{{ - m_{1} L}} } & { - m_{2} \beta_{2} e^{{ - m_{2} L}} } & { - m_{3} \beta_{3} e^{{ - m_{3} L}} } & {m_{1} \beta_{1} e^{{m_{1} L}} } & {m_{2} \beta_{2} e^{{m_{2} L}} } & {m_{3} \beta_{3} e^{{m_{3} L}} } \\ \end{array} } \right]\left\{ {\begin{array}{*{20}c} {C_{1} } \\ {C_{2} } \\ {C_{3} } \\ {C_{4} } \\ {C_{5} } \\ {C_{6} } \\ \end{array} } \right\} = \left\{ {\begin{array}{*{20}c} 0 \\ 0 \\ 0 \\ 0 \\ {\overline{G}(s)} \\ 0 \\ \end{array} } \right\}.$$

The above linear equation system provides the unknown parameters $C_{i}$ and $C_{i + 3}$. The solution in the transform domain of Laplace is thus complete.

## Inversion of the Laplace transforms

The Riemann-sum approximation technique is employed to get numerical results for the purposes of determining the temperature, the displacement, bending moment and the stress distributions in the time domain. Any functions in the Laplace domain can be converted to the time domain using the following relation^38^

56 $$f\left( t \right) = \frac{{e^{\zeta t} }}{t}\left[ {\frac{1}{2}Re\left[ {\overline{F}\left( \zeta \right)} \right] + Re\mathop \sum \limits_{n = 0}^{N} \left( {\overline{F}\left( {\zeta + \frac{in\pi }{t}} \right)\left( { - 1} \right)^{n} } \right)} \right]$$

where ${\text{Re}}$ and $i$ are the real part and the imaginary number unit. For faster convergence, numerical experiments have shown that the value that satisfies the above relation is $\zeta \simeq 4.7/t$^7^.

Sample transform functions. To show the degree of precision, the findings of our numerical inversion approach are compared with the accurate estimates.

We will check two examples to illustrate the applicability of the suggested numerical inversion technique. We take the transformed function $\overline{{g_{1} }} \left( s \right) = \frac{1}{{1 + s + s^{2} }}$ and $\overline{{g_{2} }} \left( s \right) = \frac{{{\text{exp}}\left( { - 1/s} \right)}}{{s^{3/2} }}$ which are the transforms of the functions $g_{1} \left( t \right) = \left( {2/\sqrt 3 } \right){\text{exp}}\left( { - t/2} \right)\sin \left( {\sqrt 3 t/2} \right)$ and $g_{2} \left( t \right) = \frac{{\sin \left( {2\sqrt t } \right)}}{\sqrt \pi }$. To invert the transform $\overline{{g_{1} }} \left( s \right)$, the parameter values of^6^ were used, namely $\zeta = 0.421$, $N = 19$ and $t_{1} = 7.5$^39,40^. Table 1 displays the numerical disparities between numerical values and exact values of $g\left( t \right)$ for the four approaches for time values ranging from 0.0 to 10.0. Comparison with the exact inverse Laplace transform shows a fairly good approximation.

**Table 1:** Comparison between the numerical inversion and the exact inverse Laplace transform.

$t$	$g_{1} \left( t \right)$		$g_{2} \left( t \right)$	
	Exact values	Computed values	Exact values	Computed values
1	0.5335070	0.5335090	0.513016	0.513018
2	0.4192800	0.41928100	0.173811	0.173811
3	0.1332430	0.13324300	− 0.178818	− 0.178819
4	− 0.0495299	− 0.04953000	− 0.426980	− 0.426981
5	− 0.0879424	− 0.08794260	− 0.547985	− 0.547986
6	− 0.0508923	− 0.05089240	− 0.554397	− 0.554398
7	− 0.00764371	− 0.00764373	− 0.472197	− 0.472199
8	0.01271510	0.01271510	− 0.330715	− 0.330715
9	0.01280470	0.01280470	− 0.157643	− 0.157644
10	0.00538548	0.00538549	0.0233339	0.0233339

The enhanced approach can quickly achieve great accuracy without requiring large calculations. In addition, as compared to other algorithms with comparable high accuracy, the programming effort for the approach is modest^39,40^. More importantly, by increasing processing effort by a linear amount, our inversion approach may make large improvements in accuracy. Our technique is thought to work well for a broader variety of functions.

## Numerical results

In order to demonstrate the previous analysis and to compare the theoretical results obtained in the previous sections, we now take a numerical example to give computational results. Silicon, as an anthropic substance, is the material chosen for this numerical evaluation. Thus, the physical properties of the problem are expressed in SI units as^15,16^

$$\begin{aligned} & E = 169\,{\text{GPa}}, \rho = 2330\, {\text{kg}}/{\text{m}}^{3} ,C_{E} = 713\,{\text{J}}/\left( {{\text{kg}}\,{\text{K}}} \right), \\ & \alpha_{T} = 2.59 \times 10^{ - 6} \,{\text{K}}^{ - 1} , v = 0.22,{\text{ K}} = 156\,{\text{W}}/\left( {{\text{kg}}\,{\text{K}}} \right), \\ & T_{0} = 293\,{\text{K}}, \mu_{0} = 1.26 \times 10^{ - 6} \,{\text{H}}\,{\text{m}}^{ - 1} ,{ }H_{x} = 10^{7} \,{\text{A}}\,{\text{m}}^{ - 1} \\ \end{aligned}$$

The nanobeam aspect ratios are taken as $L/h = 10$ and $b/h = 0.5$. To study the effect of a non-local parameter ${\overline{\xi }}$, the values ${\overline{\xi }} = 10^{6} \xi$ is considered. The figures were prepared for $L = 1$, $t = 0.12$ and $z = h/3$ in the wide range $0 \le x \le 1$. Through the Laplace inversion, the distributions of temperature, bending moment, deflection and axial displacement with respect to the $x$ and $z$ directions are transformed to the physical domain using the relation Eq. (56). Mathematica software is used in all numerical arithmetic operations.

### Comparison investigation

In this part, a comparison study will be carried out to assess the reliability and validity of the findings, as well as the correctness of the suggested model. Using Laplace transforms, the analytical formulae for all of the distinct physical fields were recovered in the preceding section. The Riemann-sum approximation approach is used to get numerical results. Table 1 compares the non-dimensional physical fields (temperature ${\varvec{\theta}}$ and deflection ${\varvec{w}}$) of nanoscale beams with previously published results^15^. In this case $\overline{\user2{\xi }} = 0.003$, ${\varvec{\tau}}_{{\varvec{q}}} = 0.2$, ${\varvec{\tau}}_{{\varvec{\theta}}} = 0.1$ and ${\varvec{t}}_{0} = 0.1$, and ${\varvec{H}}_{{\varvec{x}}} = 0$. When these findings were compared to those obtained from literary works^15^, it was observed that the behavior of mechanical and thermal waves of varying magnitudes is relatively comparable.

The existence of non-local operator, ramping time and phase lag parameters in the behavior of thermal and mechanical waves is indicated by the data shown in the table. The findings of the current technique and those of Ref.^15^ are in good agreement, suggesting that our model is valid, as shown in Table 2.

**Table 2:** Comparison of the values of temperature $\theta$ and deflection $w$ with Ref.^15^.

$x$	Temperature $\theta$		Deflection $w$	
	Present	Ref.^15^	Present	Ref.^15^
0	0.503701	0.522177	0	0
0.1	0.442488	0.459868	− 0.00434670	− 0.00429421
0.2	0.389772	0.406197	− 0.00817799	− 0.00807736
0.3	0.344908	0.360505	− 0.01111450	− 0.01097540
0.4	0.307294	0.322179	− 0.01291190	− 0.01274870
0.5	0.276389	0.290676	− 0.01344720	− 0.01327700
0.6	0.251732	0.265529	− 0.01270640	− 0.01254670
0.7	0.232942	0.246361	− 0.01077450	− 0.01064100
0.8	0.219729	0.232877	− 0.00782633	− 0.00773114
0.9	0.211883	0.224869	− 0.00412118	− 0.00407196
1	0.209281	0.222214	0	0

**Figure 2:**
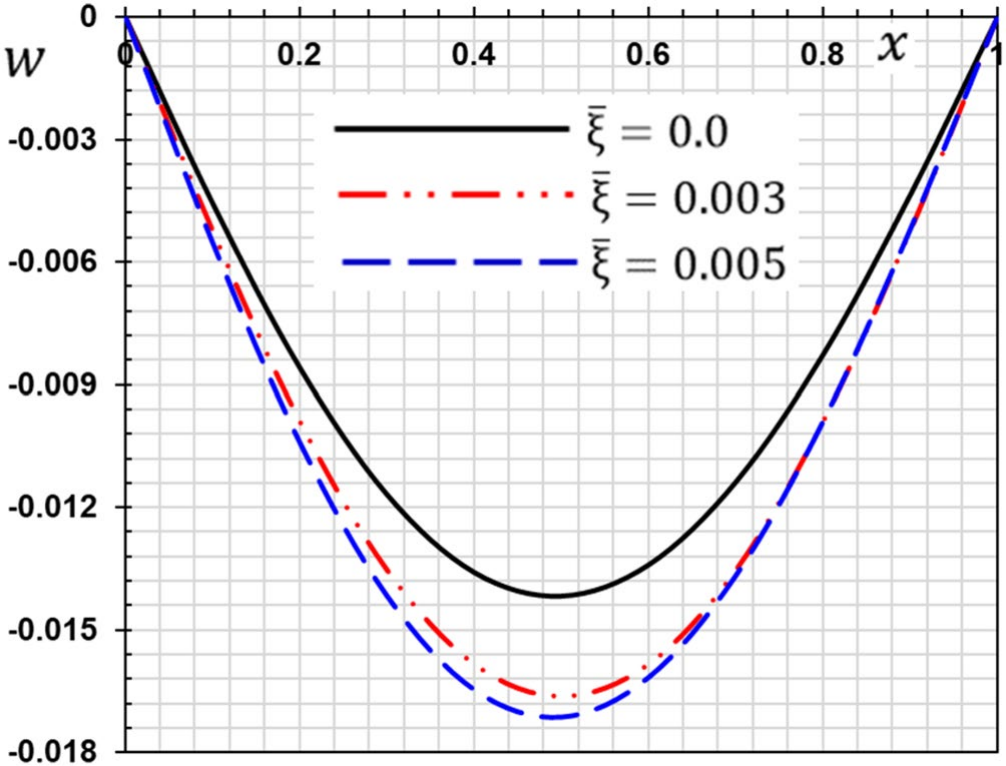
The transversal deflection $w$ with the non-local parameter ${\overline{\xi }}$.

**Figure 3:**
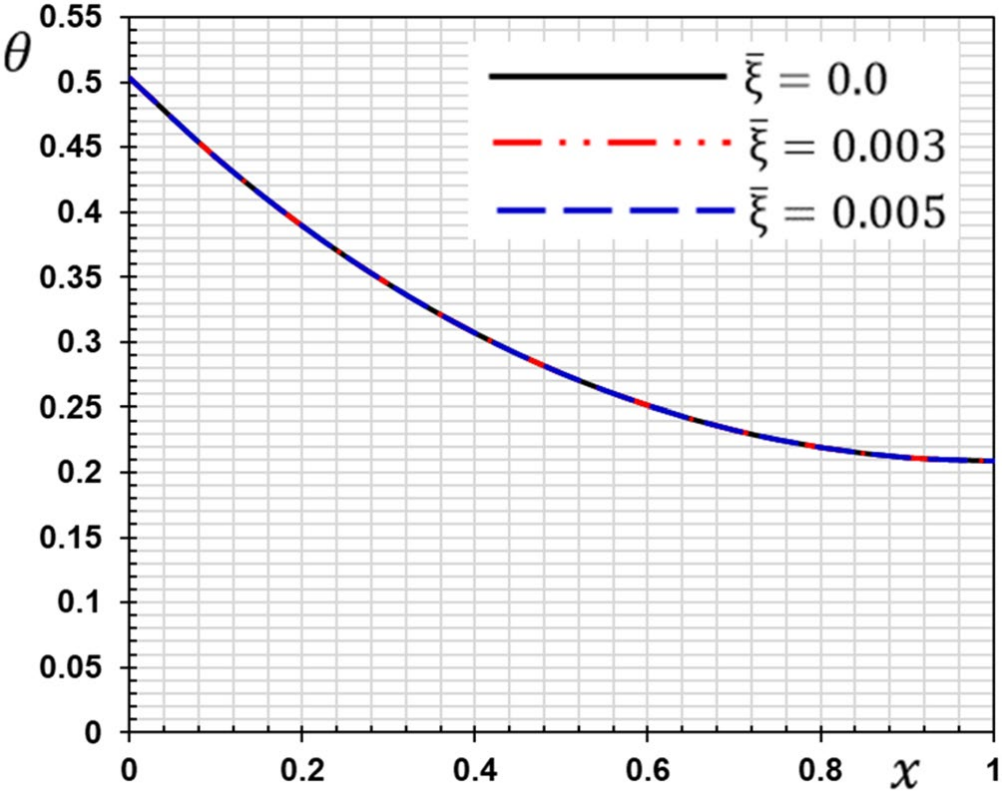
The temperature $\theta$ with the non-local parameter ${\overline{\xi }}$.

**Figure 4:**
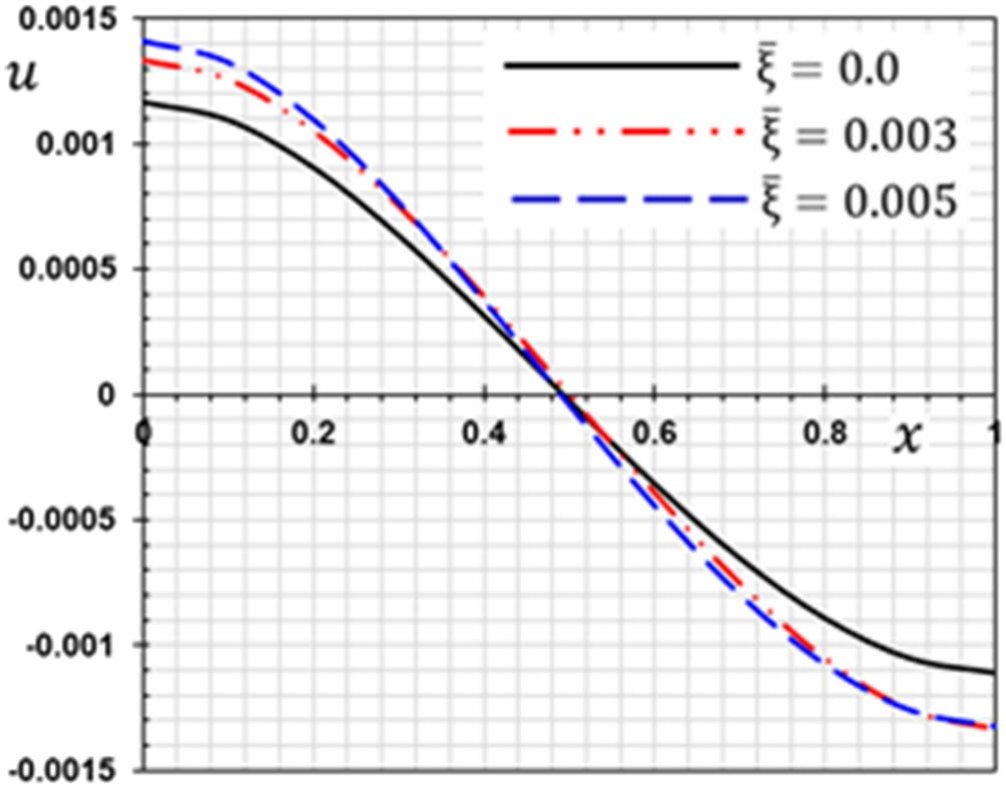
The displacement $u$ with the non-local parameter ${\overline{\xi }}$.

**Figure 5:**
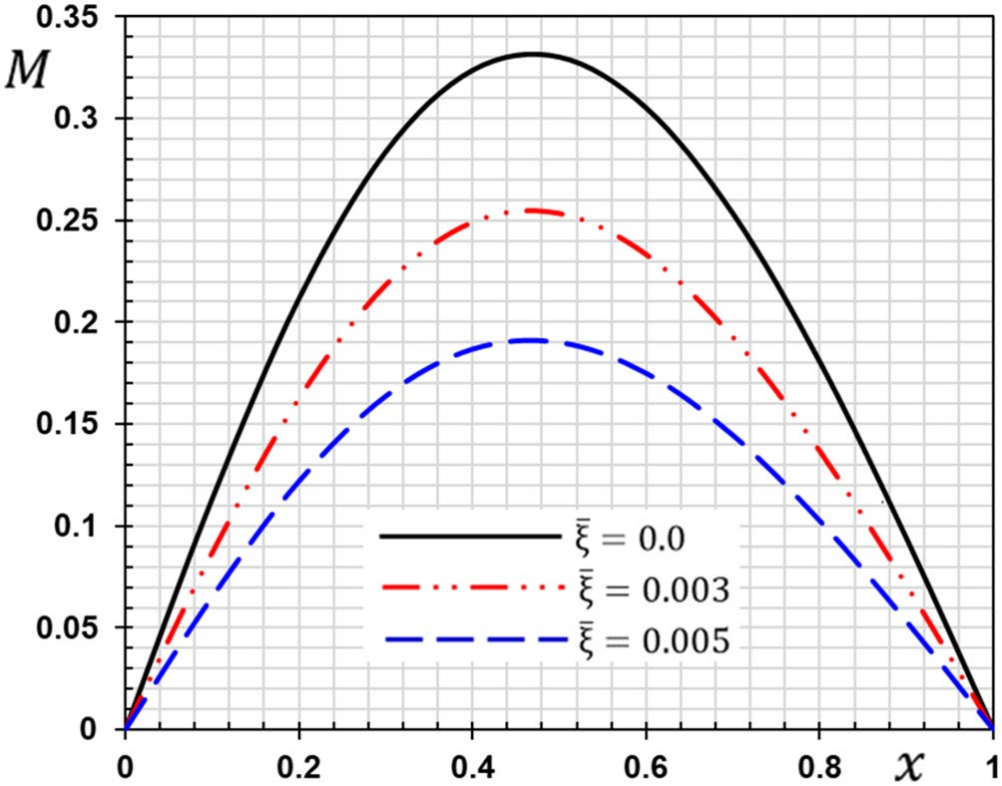
The bending moment $M$ with the non-local parameter ${\overline{\xi }}$.

**Figure 6:**
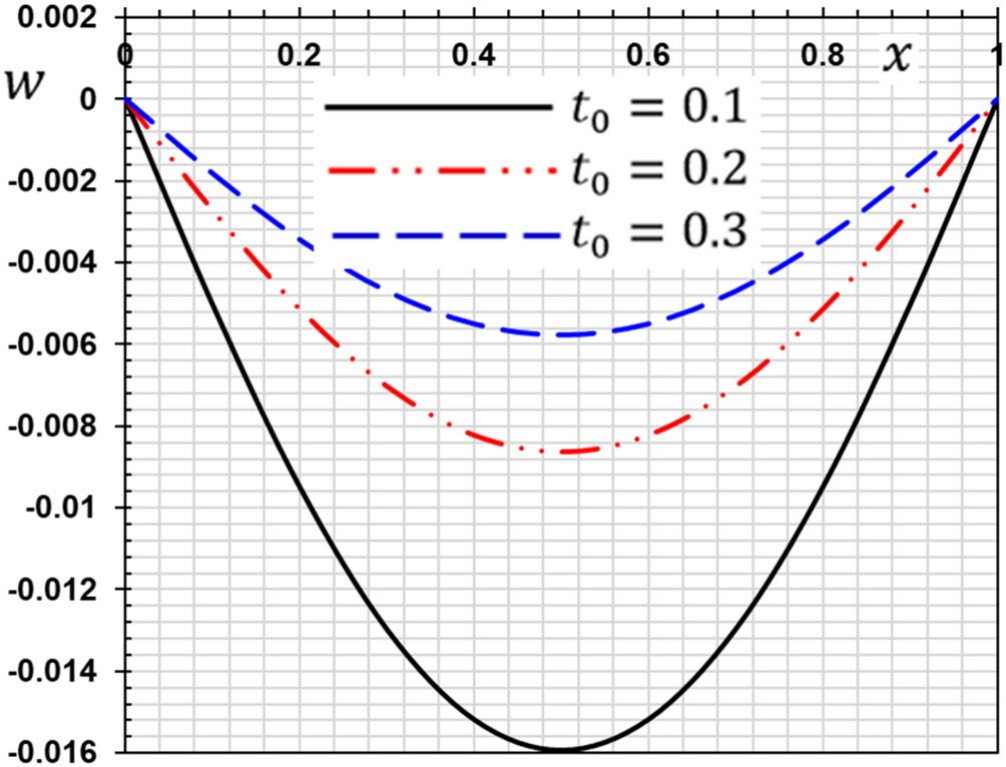
The influence of ramping time $t_{0}$ on the transverse deflection.

**Figure 7:**
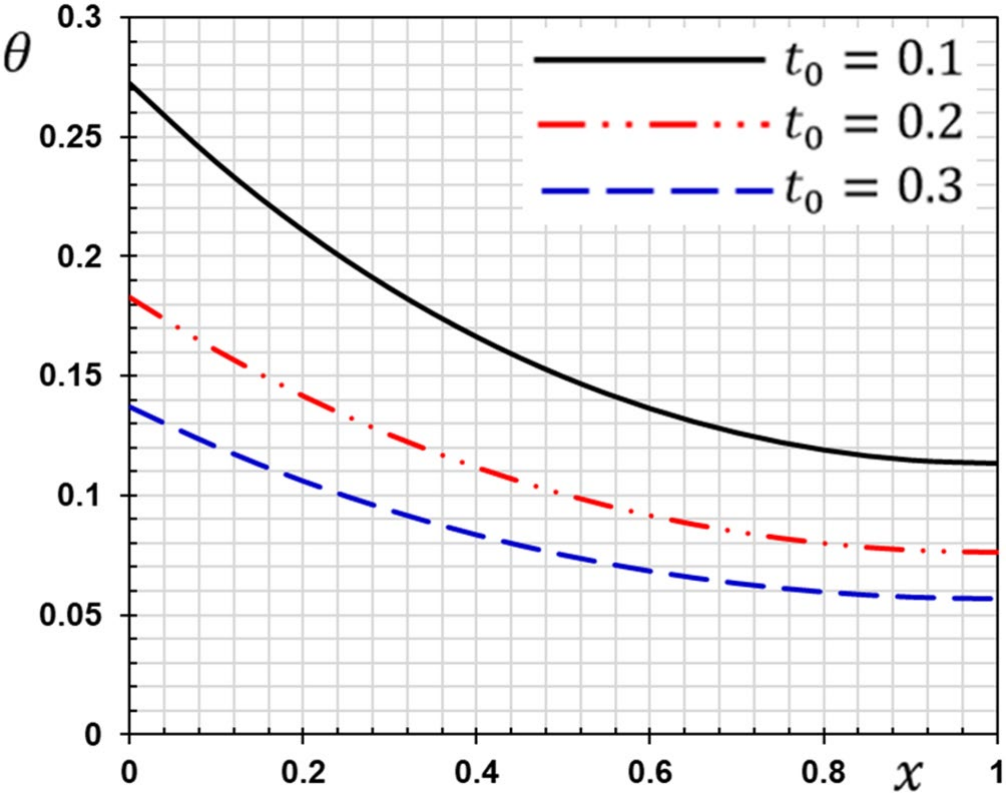
The influence of ramping time $t_{0}$ on the temperature.

**Figure 8:**
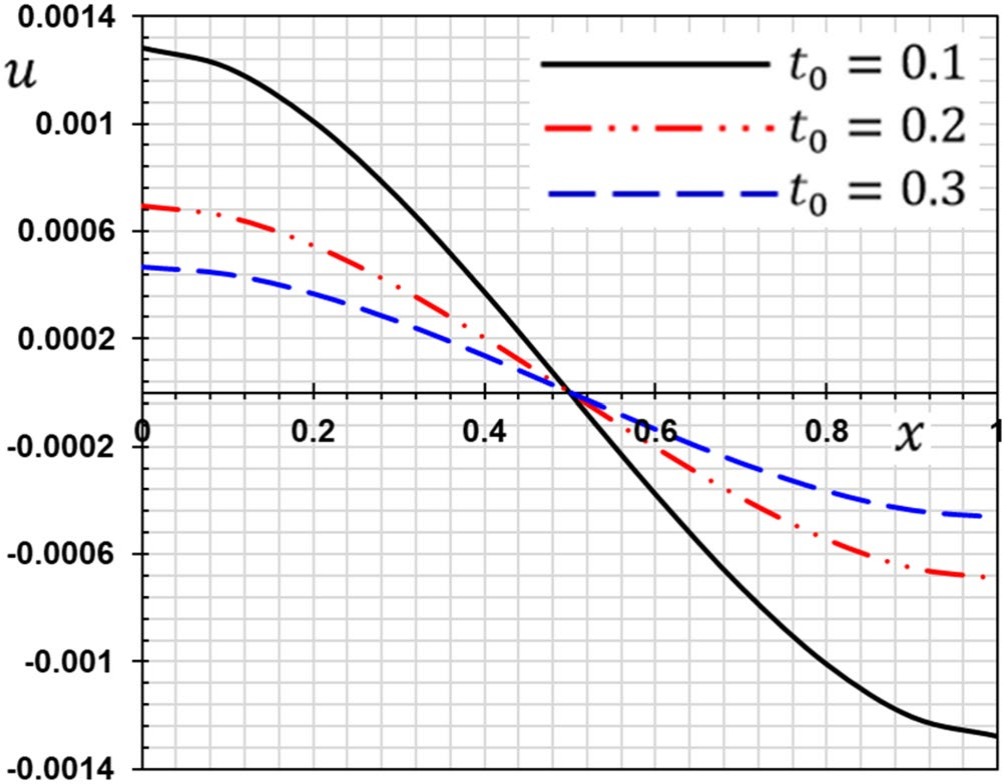
The influence of ramping time $t_{0}$ on the displacement.

**Figure 9:**
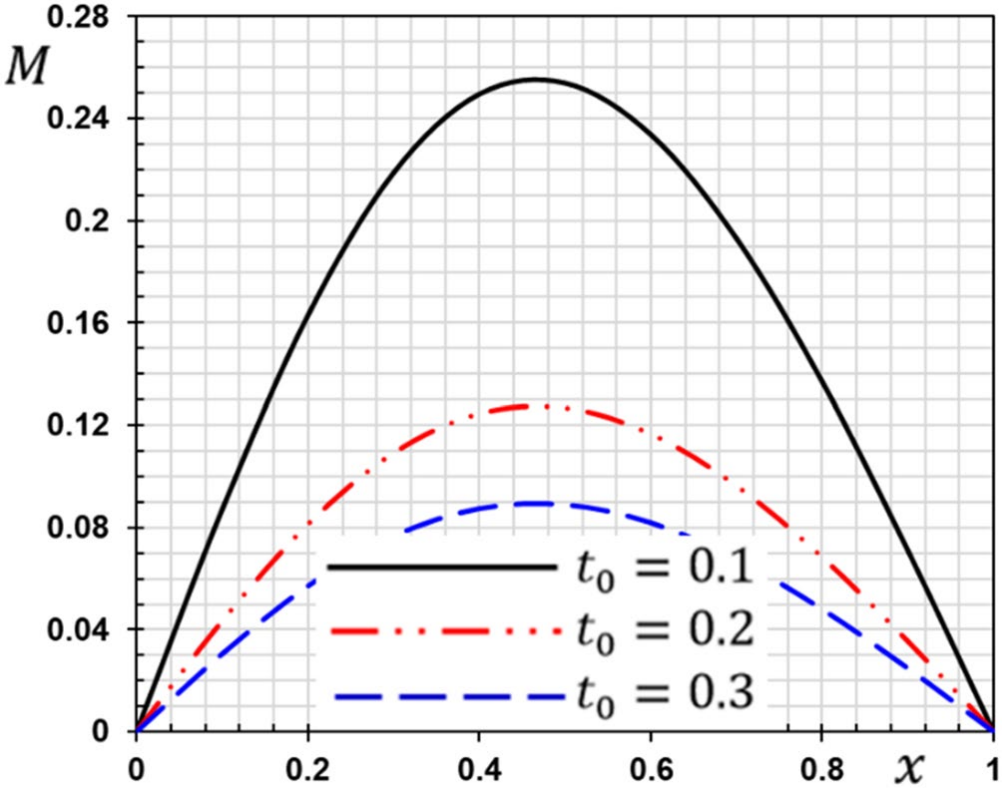
The influence of ramping time $t_{0}$ on the flexure moment.

**Figure 10:**
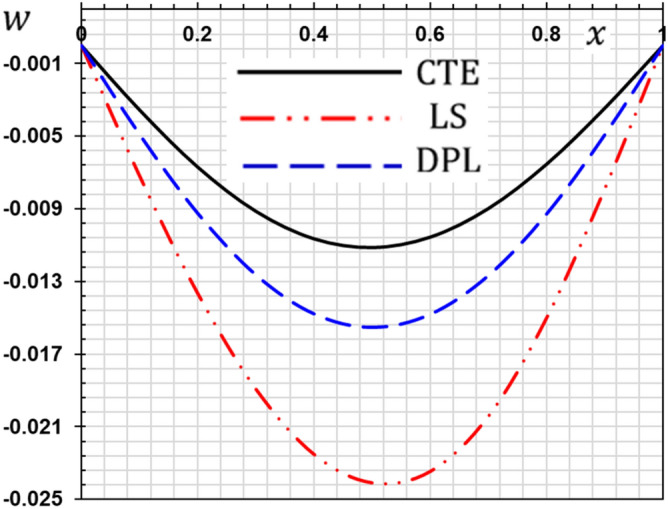
The transverse deflection with different thermoelastic models.

**Figure 11:**
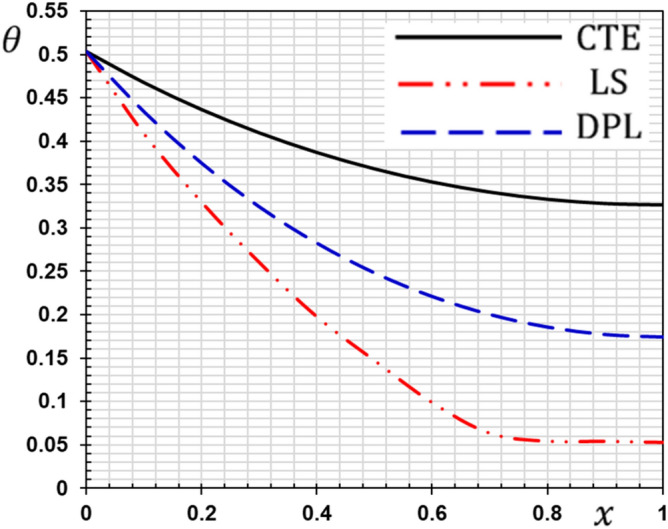
The temperature with different thermoelastic models.

**Figure 12:**
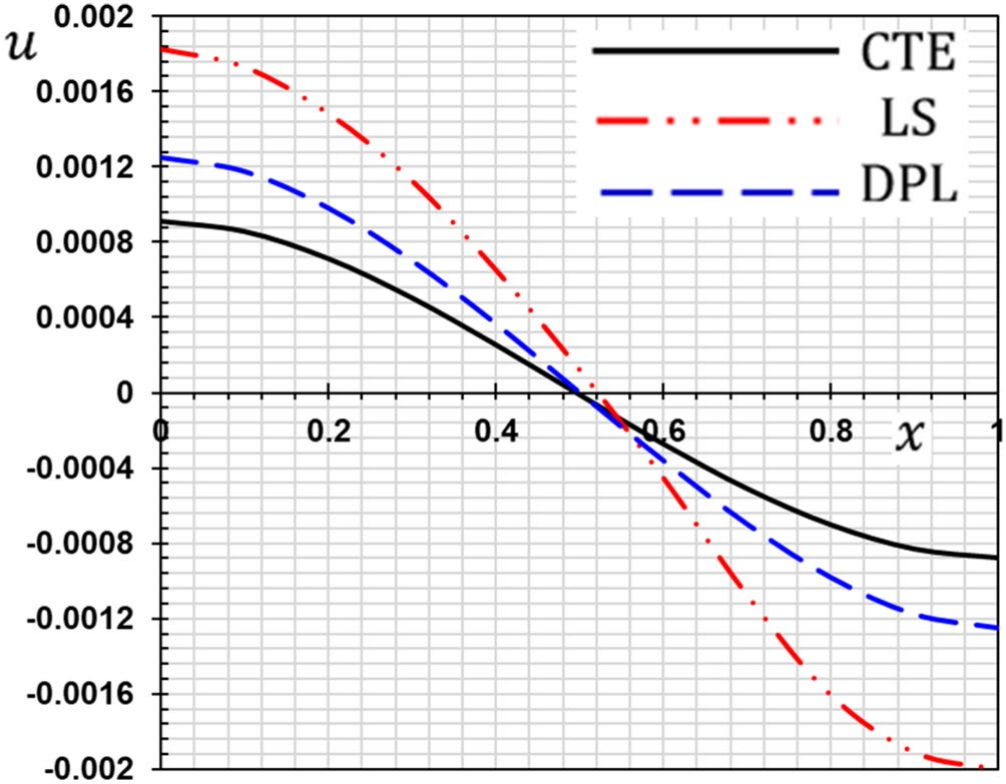
The displacement with different thermoelastic models.

**Figure 13:**
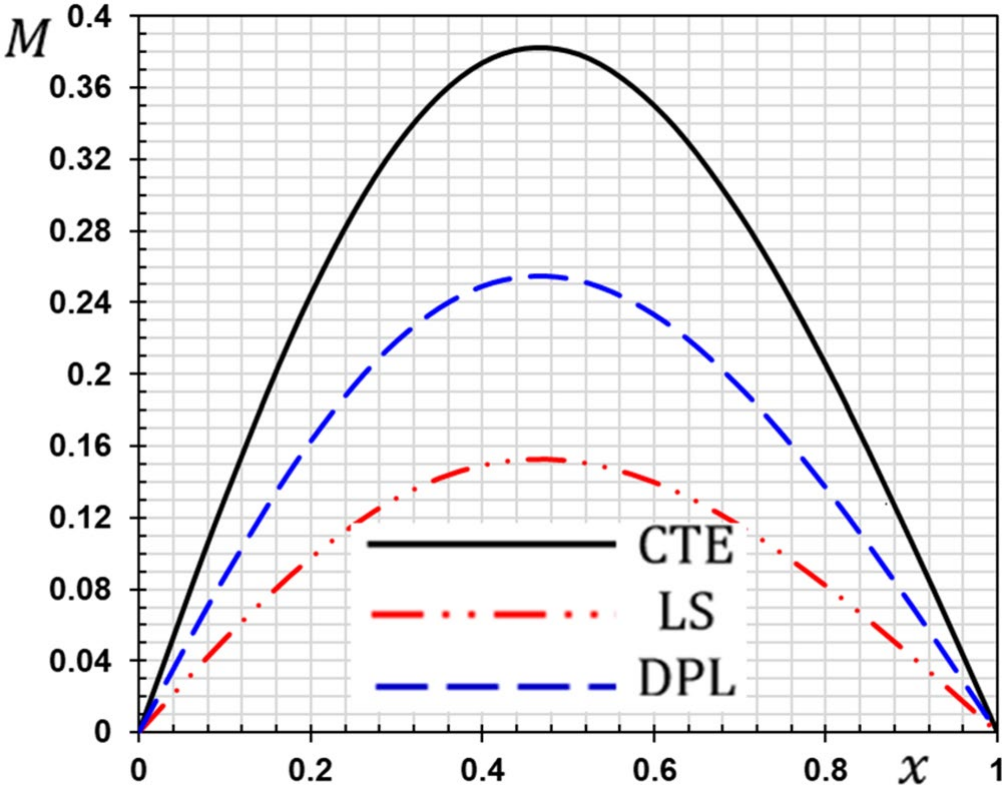
The flexure moment $M$ with different thermoelastic models.

These numerical calculations are made in the following four cases:

- **Case I:** Explore how the values of different non-local parameter ${\overline{\xi }}$ values vary with the thermodynamic temperature, dimensionless deflection, displacement and bending moment (see Figs. 2, 3, 4, 5). In this case, we take $t_{0} = 0.1$ and assume that the PLs $\tau_{q}$ and $\tau_{\theta }$ are assumed to be constants $\left( {\tau_{q} = 0.2, \tau_{\theta } = 0.1} \right)$.
- **Case II:** Explain how the thermophysical fields change by varying the values of the different ramping time parameter $t_{0}$ when taking a constant value of the non-local parameter ${\overline{\xi }} = 0.003$ (Figs. 6, 7, 8, 9).
- **Case III:** A comparison is studied between the different thermoelastic models in the event that the values of the ramping time $t_{0}$ and the non-local ${\overline{\xi }}$ coefficients are held constant (Figs. 10, 11, 12, 13).
- **Case IV:** It shows the effect of the initial magnetic field coefficient $H_{x}$ on the change of all the variables of the studied physical fields $\left( {w, \theta , u, M} \right)$.

### Case I: The effect of the nonlocal parameter

The non-local theorem is proved by replacing the variables in the standard continuous medium with nonlocal coefficients and expressing internal forces as nonlocal expressions. When looking for mechanical behavior at the micro/nano-scale, other physical phenomena must also be taken into account. The physical qualities of the structure, as well as the possibilities of deformation, are influenced by physical events. At nanometer scales, the size effect is often prominent and its effect on the mechanical performance of the nanostructures must be explicitly addressed. Thus, magneto-electro-elastic nanostructuring has become an active research project that depends on its size. For this reason, we will study the effect of the nonlocal parameter on the distributions of different fields.

In this case, three values for the non-local parameter ${\overline{\xi }} = 0$ are taken into consideration. For the nonlocal theory, we will take ${\overline{\xi }} = 0.003$ and ${\overline{\xi }} = 0.005$ while in the classical theory of elasticity we put ${\overline{\xi }} = 0$. The values of the other factors will be fixed as $\tau_{q} = 0.2$, $\tau_{\theta } = 0.1$ and $t_{0} = 0.1$. The numerical values of the different domains are represented in Figs. 2, 3, 4 and 5.

From the figures, the following important conclusions and notes can be written:

- As reported by^41^, small-scale effects play a significant impact in the thermal and mechanical properties and responses of micro/nanostructures, according to the results of experiments. As a result, nonlocal influences should be addressed while analyzing the mechanical behavior of nanostructures.
- It is clear from Fig. 2 that the s lateral vibration $w$ satisfies the boundary conditions as it always starts and ends with zero values at both edges of the nanobeam.
- The gap between non-local and classical theories increases with the increase of the non-local parameter.
- The non-local parameter has a significant effect on the lateral vibration $w$ change. With the increase of the non-local parameter ${\overline{\xi }}$ the amount of deflection $w$ increases and thus the stiffness of the nanobeam decreases. This is the case because the stiffness of the overall beam has been reduced.
- The decrease in deflection is more evident at higher levels and should not be disregarded. In summary, the small scale influence makes the nanobeam more flexible because elastic springs connect the atoms in nonlocal theory.
- The maximum deflection occurs at the center of the beam ($x = L/2$) while the ends meet the stipulated boundary criteria. The largest deflection that occurs in the middle of the beam may be due to the simply supported boundary conditions.
- The curve representing the shape of the temperature change of the beam for certain non-local parameters is given in Fig. 3. It is clear from Fig. 3 that the non-local coefficient has little effect on the change of temperature $\theta$ distribution as reported by^37^.
- Figure 3 shows that the temperature $\theta$ gradually decreases in the direction of wave propagation away from the first edge as the distance $x$ increases.
- With and without nonlocality impact, Fig. 4 shows how the displacement $u$ varies as a function of both time and distance *x*. The displacement begins with positive values at the first edge and gradually lowers until it becomes negative and inverted at $x = 0.5$.
- The crossing phenomena in displacement were demonstrated to be dependent on the linear coupling stiffness between the layers, as well as the nonlocal parameter.
- Fig. 4 shows that this parameter affects the displacement field considerably. The waves achieve the constant state according to the non-local value of parameter ${\overline{\xi }}$. The amplitude of displacement $u$ is increasing with ${\overline{\xi }}$, as shown in Fig. 4.
- In Fig. 5, the bending moment $M$ fades out at the first beam boundary $x = 0$ and after a certain distance, it reaches a local maximum value. It decreases finally to zero. From Fig. 5 it is clear that the bending moment is reduced by the parameter ${\overline{\xi }}$.
- Figure 5 shows that tiny size has a big impact on the bending moment $M$ but fades away as the beam gets longer.
- The moment $M$ reduces as the coefficient increases, implying that the nonlocal beam moment is smaller than the local equivalents.
- The nonlocal parameter may thus be set appropriately to minimize the bending moment values and ensure that the nanobeam behavior is in the elastic region.
- Because in nonlocal theory, elastic springs connect the atoms, the small scale effect makes the beam more flexible^25,42^.
- Since the classical continuum theory does not account for nonlocality effects when modeling materials, nonlocality-dependent continuity theories must be used.
- The figures indicate that the tiny scale has a major influence on short nanobeams, and that as the beam length increases, the influence gradually decreases. As a result, the tiny size will be reduced to produce a long skinny nanobeam.

### Case II: The effect of the ramping time parameter

The time it takes the heated material to achieve the desired temperature is known as the ramp-up time. In other words, the time it takes for the heated medium to reach the target temperature is known as the ramp-up time. Once the material heating system has reached the required temperature, it will just need to cycle on and off to keep it there, consuming far less energy than during the initial ramp-up period. For aerospace constructions, the temperature ramp rate is important for modeling real-world temperature changes.

The second case studies how the non-dimensional deflection, thermodynamic temperature, displacement and bending moment of the nanobeam varies with the time gradient parameter (ramping time) $t_{0}$. In the calculations, the phase lags (PLs) $\tau_{q}$ and $\tau_{\theta }$ and the nonlocal ${\overline{\xi }}$ parameter persist constantly. Figures 6, 7, 8 and 9 are shown to examine the effect of parameter $t_{0}$ on the different distributions of the studied field variables.

From the figures, we can see the major influence on all the fields studied of the ramping time $t_{0}$ parameter. The high value of the parameter $t_{0}$ decreases in the magnitudes of all studied field variables, which can be seen very clearly at the peaks of the curves. The effect of ramping time heating on the deflection may be seen clearly in Fig. 6. The magnitudes of the deflections decrease with the increase of the heating coefficient of the ramping time $t_{0}$. Figure 7 displays the temperature changes for the different values of the heating coefficient for the ramping time $t_{0}$. We note from Fig. 7 that the temperature changes are very sensitive to the change of the heating coefficient $t_{0}$. The temperature jumps with a large decrease with the change and increase of the heating factor for the time of ramping $t_{0}$. This corresponds to the boundary condition of the problem since the temperature is inversely proportional to this parameter. The effect of the time of ramping $t_{0}$ on the displacement is shown in Fig. 8. We can see from the figure that the displacement increases with the increase of the parameter $t_{0}$ in some periods and decreases in other periods along the axis of the beam. Moreover, as shown in Fig. 9, we observed the distribution of the bending moment $M$ decreasing with the increase of the parameter $t_{0}$.

### Case III: The effect of the phase lag parameters

The third case illustrates an important comparison between the various nonlocal thermoelastic models as they can be obtained as special cases from the presented thermoelastic dual phase lag model (DPL). The classical theory of thermoelasticity (CTE) can be taken by putting the phase lag $\tau_{\theta } = 0$ and ${ }\tau_{q} = 0$. The generalized theory of thermoelasticity with a single phase-lag (LS) introduced by Lord and Shulman can be realized when the phase lag $\tau_{\theta } = 0$ and ${ }\tau_{q} = 0.01$. The flexure moment $M$, the distributions of the deflection $w$, thermodynamic temperature $\theta$ and the displacement $u$ for different models of thermoelasticity are presented graphically in Figs. 10, 11, 12 and 13.

From these figures, we show that the classical thermoelasticity theory CTE is near to the DPL model of thermoelasticity. However, The LS model distributions differ from the DPL model distributions. We observed moreover as presented in Fig. 10 the distribution of the flexure moment $M$ in the case of the CTE model is small compared to the other models. It is noticed in Fig. 6 that the temperature distribution in the case of the classical theory is higher than the distributions in the case of the other generalized models. In other words, heat waves propagate at lower speeds in the case of generalized models than in the traditional theory.

We note that due to the presence of relaxation times the speed of thermal and mechanical waves is reduced. We also note that in the case of the Tzou model^5^, there are two relaxation times (phase lags), so the reduction is greater than in the case of Lord and Shulman model^2^.

### Case IV: The effect of the initial magnetic field

**Figure 14:**
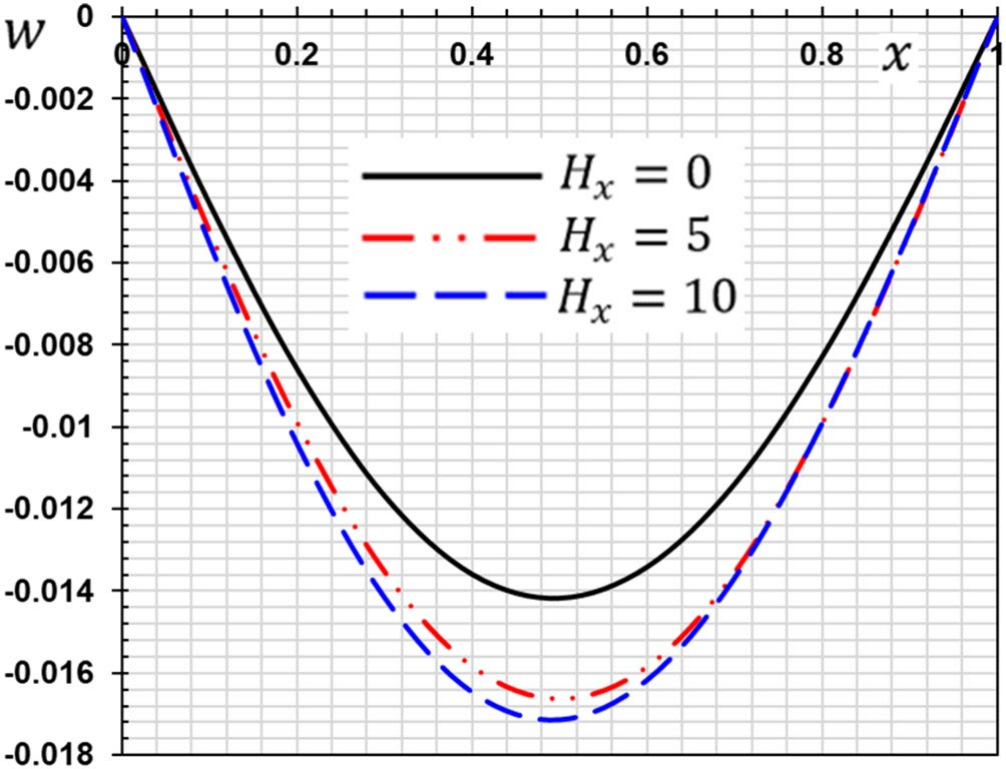
The deflection ${\text{w}}$ with the magnetic field $H_{x}$.

**Figure 15:**
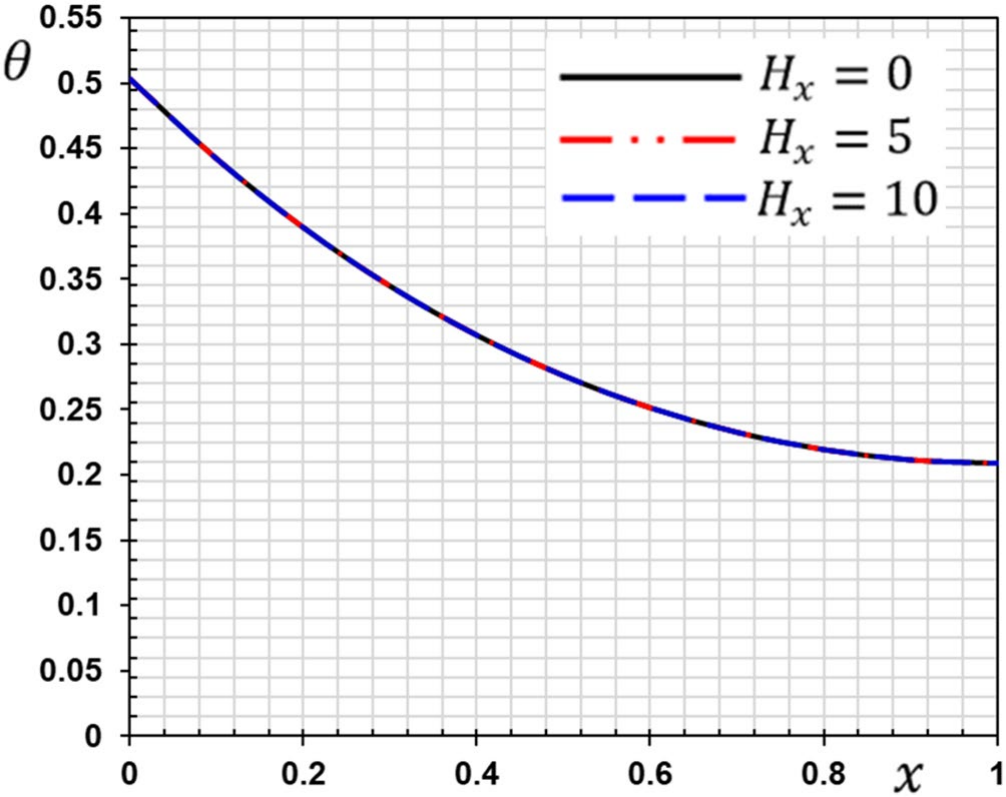
The temperature $\theta$ with the magnetic field $H_{x}$.

**Figure 16:**
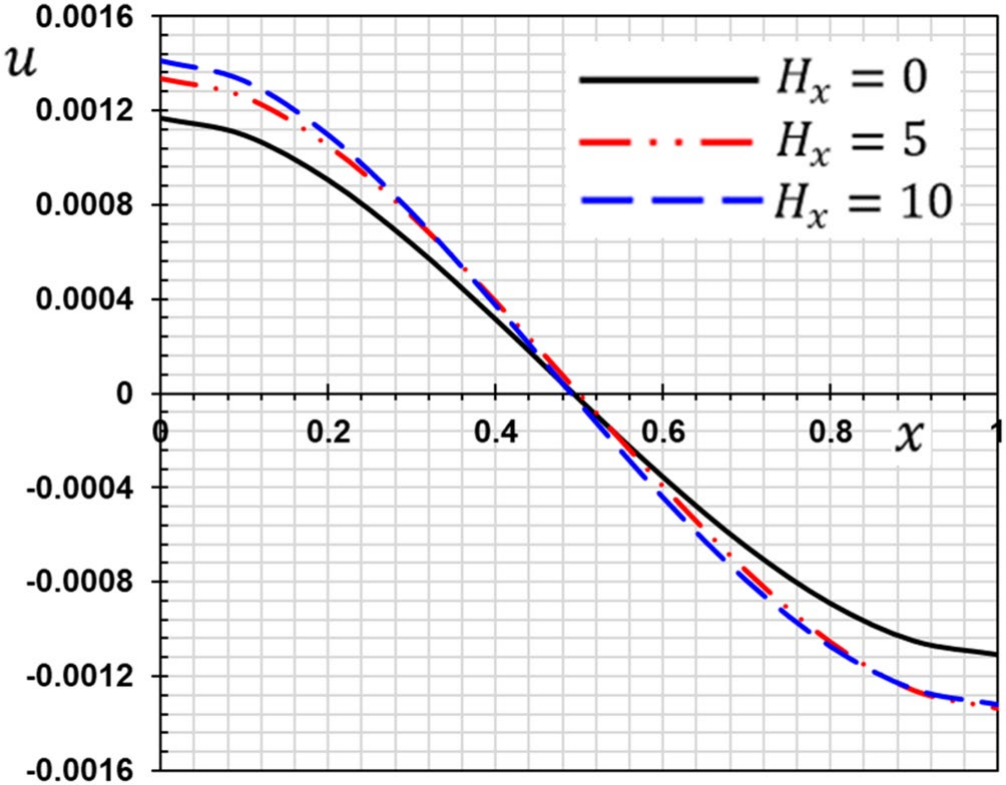
The displacement $u$ with the magnetic field $H_{x}$.

**Figure 17:**
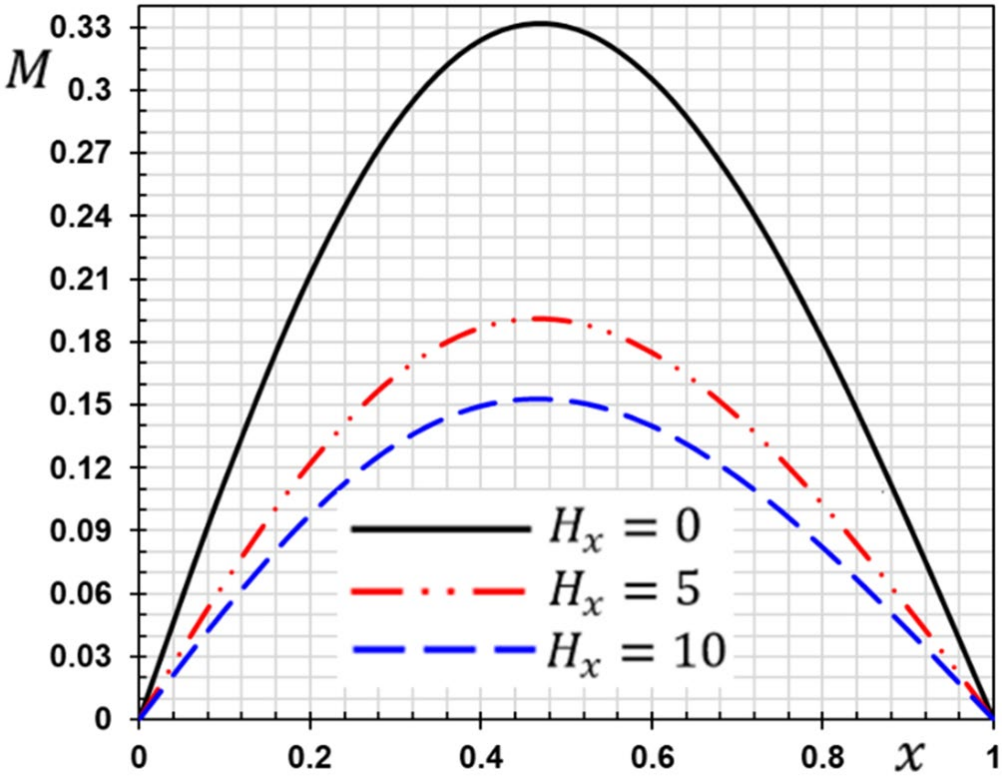
The flexure moment $M$ with the magnetic field $H_{x}$.

In the last case, we have analyzed the influence of the initial magnetic field parameter $H_{x}$ on all studied field variables ($\theta , w, u, M$) in the wide range $0 \le x \le 1$. We take two values of the initial magnetic field $H_{x} = 5,10{ }$ in the presence of the magnetic field. When $H_{x} = 0$, the absence of a magnetic field was considered. When the parameters $\tau_{q} = 0.2, \tau_{\theta } = 0.1, {\overline{\xi }} = 0.003$ and $t_{0} = 0.1$, are supposed to be constants some outcomes achieved are presented graphically in Figs. 14, 15, 16 and 17. The current findings might be beneficial in the modelling of magnetically operated nanomotors, nanostructures, and fluid-conveying nanomaterials.

The effect of the magnetic field on the responses of the nanobeam can be seen clearly in this situation. The amplitude of vibrations is significantly altered by raising the magnetic field value. The amplitude of deflection field vibration responses rises as the magnetic field increases (see Fig. 14), but the temperature does not change as the magnetic field changes (see Fig. 15). From Fig. 16, we can see that the magnitudes of the displacement $u$ rise with the rising magnetic field. As seen in Fig. 17, the amplitude of the bending moment decreases when the magnetic field is increased, demonstrating the effect of the magneto-thermoelastic field on bending moment vibration responses. When the magnetic force characteristics change from tension to compression, the bending moment falls as predicted.

## Conclusion

In this paper, thermal vibration analysis of nanobeams by considering the magneto-thermo-elastic effect was studied using the non-local elasticity of Eringen and the Euler–Bernoulli beam principle. Also, based on the two-phase delay thermoelastic model, the problem was also studied. The effect of a permanent magnetic field on the vibrations of nanobeams was modeled using the concept of the Lorentz force. The Laplace transformation method was used to calculate analytical findings for dimensionless temperature, deflection and thermal moment. Although the illustrations are self-explaining in showing the various peculiarities that occur during the wave spread, the following observations can be added.

- A significant effect was observed for the non-local response compared to the absence of the non-local effect. The non-local coefficient has a prominent role in changing the studied physical fields except for temperature.
- The maximum deflection that occurs at the center of the beam while the ends meet the stipulated boundary criteria. The largest deflection occurs in the middle of the beam may be due to the simply supported boundary conditions.
- The crossing phenomena in displacement were demonstrated to be dependent on the linear coupling stiffness between the layers, as well as the nonlocal parameter.
- The wave propagation characteristics of nanobeams are affected by nonlocal scale parameter, temperature change, thermal load, and external magnetic potential, according to numerical results.
- All physical fields within the body are strongly affected by changing ramping time parameter. The temperature ramp rate can be used to model real-world temperature variations in aerospace structures.
- The variation of the field quantities is shown to have a significant effect on thermal-phases lag parameters. It is shown that in conventional thermoelasticity the profile of the physical field quantity is larger than in generalized models.
- These results obtained in this paper are important for the design of integrated functional devices and smart devices.
- The current work only analyses the nanostructure exposed to longitudinal magnetic field; in the future, it would be helpful to observe the nanostructure exposed to magnetic field in 3-dimensional directions.

## Data Availability

Data will be provided on request.
